# Arginine deprivation affects glioblastoma cell adhesion, invasiveness and actin cytoskeleton organization by impairment of β-actin arginylation

**DOI:** 10.1007/s00726-014-1857-1

**Published:** 2014-11-02

**Authors:** Iuliia Pavlyk, Yuriy Rzhepetskyy, Adam K. Jagielski, Jakub Drozak, Anna Wasik, Galyna Pereverzieva, Marta Olchowik, Leoni A. Kunz-Schugart, Oleh Stasyk, Maria Jolanta Redowicz

**Affiliations:** 1 Nencki Institute of Experimental Biology, Polish Academy of Sciences, 3 Pasteur St., 02-093 Warsaw, Poland; 2 Institute of Cell Biology, National Academy of Sciences of Ukraine, 14/16 Drahomanov St., Lviv, 79005 Ukraine; 3 Department of Metabolic Regulation, Faculty of Biology, Institute of Biochemistry, University of Warsaw, Miecznikowa 1, 02-096 Warsaw, Poland; 4 OncoRay- National Center for Radiation Research in Oncology, Medical Faculty and University Clinic Carl Gustav Carus, Technische Universität Dresden, and Institute of Radiobiology, Helmholtz-Zentrum, 01307 Dresden, Germany

**Keywords:** Amino acid deprivation, Actin cytoskeleton, Actin arginylation, Cancer, Invasiveness, Cell migration

## Abstract

A deficit of exogenous arginine affects growth and viability of numerous cancer cells. Although arginine deprivation-based strategy is currently undergoing clinical trials, molecular mechanisms of tumor cells’ response to arginine deprivation are not yet elucidated. We have examined effects of arginine starvation on cell motility, adhesion and invasiveness as well as on actin cytoskeleton organization of human glioblastoma cells. We observed for the first time that arginine, but not lysine, starvation affected cell morphology, significantly inhibited their motility and invasiveness, and impaired adhesion. No effects on glia cells were observed. Also, arginine deprivation in glioblastoma evoked specific changes in actin assembly, decreased β-actin filament content, and affected its N-terminal arginylation. We suggest that alterations in organization of β-actin resulted from a decrease of its arginylation could be responsible for the observed effects of arginine deprivation on cell invasiveness and migration. Our data indicate that arginine deprivation-based treatment strategies could inhibit, at least transiently, the invasion process of highly malignant brain tumors and may have a potential for combination therapy to extend overall patient survival.

**Electronic supplementary material:**

The online version of this article (doi:10.1007/s00726-014-1857-1) contains supplementary material, which is available to authorized users.

## Introduction

Tumor cells often exhibit metabolic defects in amino acid biosynthesis and, concomitantly, hypersensitivity to deprivation of certain amino acids. This feature was exploited to develop enzymotherapies based on single amino acid deprivation, such as asparagine, methionine and arginine (Covini et al. 2012; Agrawal et al. 2012; Delage et al. 2010). Still, the molecular mechanisms determining the sensitivity of malignant cells to single amino acid deficiency remain a subject of ongoing investigations (Bobak et al. 2010; Morrow et al. 2013).

Arginine is a semi-essential amino acid in humans and its physiological level is maintained through protein degradation, endogenous synthesis and food intake (Morris 2007). It has been established that an increased dependence on exogenous arginine is typical for many malignant tumor cells, both in vitro and in vivo (Scott et al. 2000; Wheatley et al. 2005; Feun et al. 2008). Also, a growing number of tumors is being identified as deficient in arginine anabolic enzyme argininosuccinate synthetase (ASS) and, thus auxotrophic for arginine (Allen et al. 2014; Delage et al. 2010; Dillon et al. 2004). Growth of such tumors can be controlled by the treatment with two arginine degrading recombinant enzymes, human arginase I or bacterial arginine deiminase; both currently undergoing phase I/II clinical trials (Glazer et al. 2010; Yang et al. 2010; Yau et al. 2013).

The elevated tumor cell sensitivity to arginine deprivation may result from the complex nature of arginine metabolism and its impact on a number of metabolic and signaling pathways. We recently showed that in vitro arginine is essential for the growth of malignant cells of different organ origin but the levels of intrinsic arginine metabolic enzymes do not determine tumor cell response to arginine-deprivation stress (Bobak et al. 2010). We also observed that the sensitivity of tumor cells to arginine starvation dramatically decreased in 3-D spheroids relative to corresponding monolayer cultures but some effects are qualitatively maintained (Vynnytska-Myronovska et al. 2012).

Although arginine deprivation-based enzymotherapy is at the developmental stage, it clearly bears a potential as an efficient, selective and relatively non-toxic approach against highly malignant ASS-negative tumors, e.g. bladder (Allen et al. 2014), melanoma (Yoon et al. 2013), pancreatic (Bowles et al. 2008), prostate (Kim et al. 2009), renal (Yoon et al. 2007) carcinomas and glioblastomas (Syed et al. 2013). For further optimization and development of rational combinational approaches, molecular signaling mechanisms governing tumor cell response to arginine deprivation have to be elucidated. Another therapeutically highly relevant question is whether and how arginine deprivation affects tumor cells motility and invasiveness. This is of particular interest in glioblastomas, the most malignant primary brain tumors that are hardly amenable to conventional therapies because of their highly motile and invasive nature (Brandes 2007). No curative treatment is available for these patients and overall survival time is rather short thus new treatment strategies are urgently needed.

In this report, we addressed this problem for the first time using three human glioblastoma cell lines as well as organotypic brain slices as experimental models. We describe the specific effects of arginine withdrawal on glioblastoma cell morphology and demonstrate that lack of arginine but not lysine profoundly impairs glioblastoma motility, adhesion and invasiveness. We propose that these effects could result from specific alterations in the actin cytoskeleton organization evoked by a deficit in β-actin arginylation.

## Materials and methods

### Cell culture and transfection

Human glioblastoma U251 MG (U251) and U87 MG (U87) cell lines were purchased from CLS Cell Lines Service (Germany) and monitored for correct genetic profile via microsatellite analyses according to a protocol described earlier (Peickert et al. 2012). LN-229 glioblastoma cells were from Prof. Marta Miączyńska from the International Institute of Molecular and Cell Biology in Warsaw, Poland. Cells were cultured at 37 °C and 5 % CO_2_ in Dulbecco’s Modified Eagle Medium (DMEM) supplemented with GlutaMAX-1, 10 % fetal bovine serum (FBS), 100 U/mL penicillin and 100 U/mL streptomycin (Gibco Live Technologies, USA). For most experiments, a formulated amino acid-free DMEM (Sigma-Aldrich, USA) was used. In order to prepare arginine- (-Arg) or lysine-free (-Lys) conditions, the medium was supplemented with all the amino acids except of arginine or lysine, respectively. A complete (control) medium was obtained by supplementation with all the amino acids (0.4 mM for arginine, and 0.8 mM for lysine and leucine). -Arg, -Lys and control media were supplemented with 5 % dialyzed FBS (Sigma-Aldrich, USA), lacking small molecules such as amino acids. U251 cells cultivated in the control medium were transfected with pmaxGFP plasmid using Nucleofection™ technology (Lonza Group Ltd., Switzerland).

### Culture conditions of astrocytes

Primary cultures of neonatal astrocytes were isolated from forebrains of 1-day-old Wistar rat pups as previously described (Zawadzka and Kaminska 2003), seeded on cover slips immediately after isolation, and cultured for 48 h in the control, -Arg or -Lys media, as described above. Housing and sacrificing procedures were performed in compliance with the European Communities Council Directive of 24 November 1986 (86/609/EEC).

### Brain organotypic slice culture

Horizontal slices (300-μm) were prepared from brains of neonatal (E13) and adult (6-month-old) female rats and cultured as described by Stoppini et al. (1991). Housing and sacrificing procedures were performed as described above.

### Growth assay

Cells (10^5^) were grown in the tested conditions and counted after 48 and 96 h using manual cell counter (Scepter 2.0, Millipore, USA), after prior trypsinization with 0.25 % trypsin–EDTA (1×) solution (Gibco Life Technologies, USA).

### MTS viability assay

On day zero, dissociated single cell suspensions were seeded in two triplicate sets of 96-well microtiter plates (10^3^ cells/well). On day 1, the supernatant of the first set of cells was replaced with fresh media and the amount of viable cells was determined after 48, 96 or 144 h using a colorimetric cell viability assay (CellTiter 96 AQueous Non-Radioactive Cell Proliferation Assay from Promega, USA). The second set was exposed to -Arg or -Lys medium for 48, 96 or 144 h, and then cultured in complete medium for additional 48-h period to assess growth restoration, followed by the viability assay performed as described above.

### Immunolocalization

Cells were treated as described in (Majewski et al. 2011), and then stained with Alexa 488-conjugated phalloidin to visualize the actin cytoskeleton. Staining for adhesive structure markers was also performed using monoclonal antibody against vinculin (Sigma-Aldrich, USA) and polyclonal antibody against talin (Santa Cruz, USA) at a dilution of 1:40. For negative controls, primary antibody was omitted. Images were collected with the Leica TCS SP5 confocal laser scanning microscope equipped with a 63× HCX oil CS UV 1.4 oil-immersion objective.

### Scanning electron microscopy (SEM)

For SEM, U251 cells were fixed in 3 % glutaraldehyde in 0.2 M phosphate buffer for 30 min at room temperature followed by 1 % OsO_4_ for 1 h, and then dehydrated through graded series of ethanol and acetone, dried by the CO_2_ critical point method and coated with a thin layer of carbon and gold. Samples were examined using a Jeol JEM-1200EX electron microscope with ASID 19 scanning attachment, operating at 80 kV.

### Random motility assay

Random motility was assessed by time-lapse microscopy as detailed previously (Majewski et al. 2011). Cells were photographed every 10 min for up to 36 h. At least 10 cells of each experimental condition were tracked (cells undergoing division or apoptosis were excluded). The tracks (reoriented to zero in migration traces) and velocity were calculated using the Metamorph software (Molecular Devices, USA).

### Transwell migration assay

The assay, based on chemotactic directional migration, was performed in 24-well Transwell inserts using 6.5-mm diameter polycarbonate filters with 8-µm pores, coated for 10 h with 30 μg/mL of type I collagen (Sigma-Aldrich, USA) as described in (Albini et al. 1987). Cells (10^4^) were placed in serum-free media into the upper chamber and allowed to migrate through the filter to the lower chamber containing DMEM media supplemented with 10 % FBS. After 6 h, non-migrated cells were removed from the upper chamber using a cotton swab. The cells, which passed through the filter and attached to its trans side were stained with 2 % crystal violet after fixation with 4 % formaldehyde. Filters were photographed and the number of migrated cells was counted.

### Invasiveness test

The effects of arginine deprivation on cell invasiveness were assessed using 8.0-μm pore size Matrigel-coated invasion chamber inserts (BD BioCoat, USA). Cells were maintained as described above and were allowed to invade through the filter for 24 h.

Also, GFP-expressing U251 cells were placed on brain organotypic slices and after 24 h the presence of fluorescent cells within a slice was examined after fixation with 4 % paraformaldehyde by means of confocal microscopy. For quantification, at least three view areas of each examined conditions of both neonatal and adult brain slices were analyzed.

### Analysis of cell adhesion

Cell adhesion to the glass surface was assessed by counting the number of vinculin-stained structures in twenty cells from each experimental condition. For analysis of cell–cell adhesion, the equal number of examined cells was plated on soft agar. The number and size of cell aggregates (with respect to the seeded cell number) were evaluated for each experimental condition.

### Western blotting

Cell lysates were separated by 12 % SDS-PAGE followed by a transfer to nitrocellulose membrane (as in 22). Actin and tubulin were detected by monoclonal antibodies against β-actin and β-tubulin (Sigma-Aldrich, USA), and against γ-actin (Millipore, USA), all diluted at 1:1,000. Talin and vinculin were detected with the respective monoclonal and polyclonal antibodies (see above) at 1:1,000 dilution. Also, CD44 was detected with anti-CD44 monoclonal antibody (R&D System, USA) and E-cadherin was detected with anti-M-cadherin antibody (BD Biosciences, UK), both at 1:1,000 dilution. Anti-mouse antibody and anti-rabbit antibody conjugated with horse radish peroxidase were applied (at 1:10,000 dilution) for detection using ECL system Pierce, USA). Protein concentration was determined by Bio-Rad protein assay reagent (Bio-Rad, USA).

### Analysis of actin organization by sedimentation assay

Cell lysates (considered as “totals” in the course of the analysis) were incubated for 1 h at 4 °C in the presence or absence of 0.04 mg/mL phalloidin (Sigma, USA). After 1-h ultracentrifugation at 150,000×*g*, the aliquots of supernatant and pellet fractions were analyzed by SDS-PAGE and Western blot to assess the amount of actin (β- and γ-isoforms) and β-tubulin. Developed blots were photographed and analyzed using the G:Box system equipped with GeneSnap and GeneTools software.

### Flow cytometric analysis of filamentous actin content

Harvested cells (10^5^) were extensively washed with ice-cold PBS and fixed in 70 % ethanol. For the analysis, the single cell suspensions were resuspended in PBS solution containing extraction buffer (0.2 M Na_2_HPO_4_, 0.1 M citric acid and 50 µg/mL RNase A (Invitrogen, USA) and incubated with Alexa488-conjugated phalloidin (0.2 U). After exhaustive washes with PBS, the fluorescence signal corresponding to F-actin was measured by flow cytometry (FACS Calibur) using CellQuest Software; 10,000 events were analyzed for each sample.

### 2D electrophoresis

2D electrophoresis was performed as described previously (Bregier et al. 2013). Cell lysates were cleared with Bio-Rad Protein Precipitation Kit as per manufacturer’s protocol. The protein mixtures were applied to ReadyStrip™ IPG 7 cm strips, pH 4–7 (linear) (Bio-Rad, USA) and then isoelectrically focused using a PROTEAN IEF cell System (Bio-Rad) followed by 12 % SDS-PAGE and a transfer onto nitrocellulose membrane and Western blotting. Actin isoelectric pattern in the lysates was determined using rabbit polyclonal anti-actin antibodies (Sigma-Aldrich, USA).

### Identification of arginylated β-actin by tandem mass spectrometry

For the analysis of samples obtained in the deprivation experiments, 40 µg of protein from each sample were separated in 12 % SDS-PAGE and silver stained as described in (Shevchenko et al. 1996). The bands corresponding to actin were excised from the gels, destained and digested with trypsin according to (Shevchenko et al. 2006). Peptides were analyzed by nanoUPLC-tandem mass spectrometry employing Acquity nanoUPLC coupled with Synapt G2 HDMS Q-TOF mass spectrometer (Waters, USA), fitted with a nanospray source and working in MS^E mode under default parameters (Drozak et al. 2013). To identify arginylated β-actin, all human protein sequences downloaded from UniProt database were supplemented with the sequences of arginylated β-actin on Arg2 and Arg3 randomized, and used as a databank of the MS/MS software. Mass chromatograms obtained from control and deprived experimental variants samples allowed us establish changes in β-actin arginylation extent. First, the chromatograms for single ion of arginylated peptide were integrated, giving a measure of the amount of arginylated peptide in each chromatographic run. However, to establish the arginylation extent, in addition to the measure of arginylated peptide present in the run, a measure of the amount of β-actin in the injected sample is also needed as a reference point. A relative measure was obtained by comparing the peak areas of five randomly chosen peptides from be β-actin on respective single ion chromatograms. The peak areas on chromatograms of samples from deprivation variants were divided by the areas of respective peaks on chromatograms from control samples and the obtained values were averaged giving a relative measure of the total amount of digested β-actin injected as a factor of the amount injected from the control sample. This factor was used to correct the peak areas of the arginylated peptide in the injection of samples coming from deprivation experiments, to the values expected if the amount of β-actin in each run was the same and to compare the values. The peak area of arginylated peptide in control was set to one and the values -Arg and -Lys were proportionally adjusted. Such an approach not only provides a common point to which a peak area of the arginylated peptide can by referred, but also compensates for possible fluctuations in the ionization efficiency, between different runs. The single ion chromatograms for selected peptides were filtered from TIC chromatogram by the software. Peptides used as reference were fragments flanked by the following amino acids in the β-actin sequence (in parentheses mass of MH+ ion): 19–28 (976.45), 40–44 in source fragment (553.26), 85–95 (1,515.75), 216–238 with carbamidomethyl modification on cysteine (2,550.17) and 239–254 (1,790.89).

### Statistical analysis

Data are presented as mean ± SD; all *p* values were calculated by two-sided Student’s *t* test. The difference was considered to be statistically significant at the level of *p* < 0.05.

## Results

### Arginine deprivation affects cell viability

First, we evaluated whether growth and viability of human U251 and U87 glioblastoma cells were affected by arginine deprivation in comparison with deprivation of lysine, an essential and also positively charged amino acid.

**Fig. 1:**
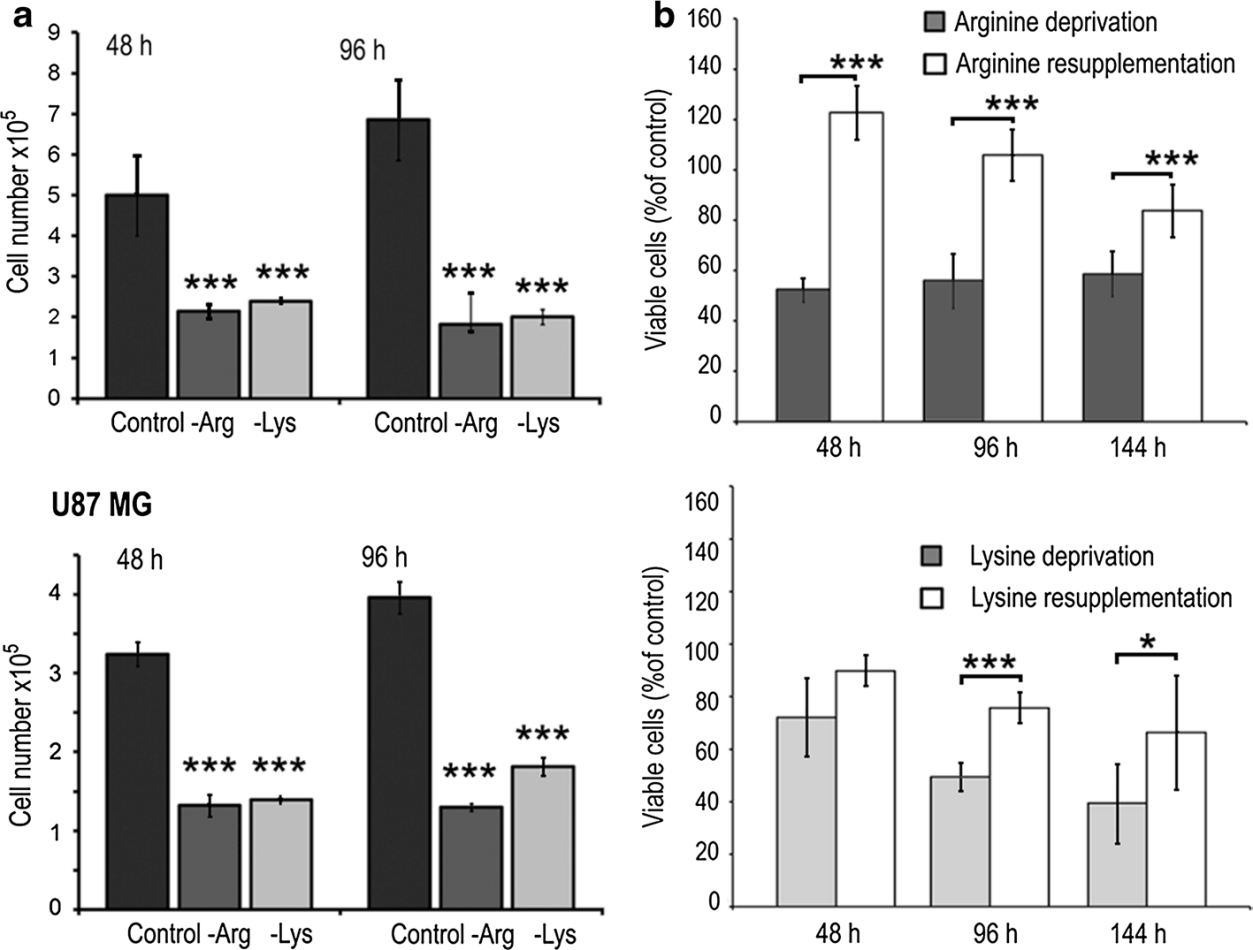
Effect of arginine deprivation on glioblastoma cells. **a** Cell growth of U251 (*upper panel*) and U87 (*lower panel*) cells cultivated in control, -Arg, -Lys and conditions. **b** U251 cell viability assessed under deprivation and re-supplementation conditions, as indicated. *Upper* and *lower panels*, arginine and lysine re-supplementation, respectively. 100 %, the number of the viable cells at time 0. Data in **a** and **b** are means ± SD; *** and *Statistical relevance *p* < 0.001 and *p* < 0.05, respectively

As expected, deprivation of arginine or lysine abrogated growth of both cell lines (Fig. 1a), indicating that both amino acids are essential in vitro. Of note, proliferation of both glioblastoma cell lines was not rescued in ornithine-, and only partially restored in citrulline-containing arginine-deficient medium, indicating the impairment of arginine biosynthesis and their full arginine auxotrophy in vitro (not shown).

Viability of -Arg cells was decreased by ~50 % with respect to control cells already after 48 h and remained on this level up to 144 h. Importantly, growth and viability of U251 cells was restored nearly to the level of control cells when cells were exposed for an additional 48 h to control medium, indicating reversibility of the inhibitory effect (Fig. 1b). Viability of -Lys cells was decreasing gradually during the time course of the experiment (up to ~40 %), and was only partially restored upon lysine re-supplementation (up to ~70 %).

Importantly, no induction of apoptosis was found in -Arg and -Lys cells as judged by the absence of activated form of PARP and a flow cytometric apoptosis test (Electronic Supplementary Material I).

### Arginine deprivation affects cell morphology

To establish whether and how single amino acid deprivation affects cell morphology, cells cultured in -Arg, -Lys or control media were stained with phalloidin to visualize filamentous actin. The staining was also performed for freshly isolated rat primary culture glia cells.

**Fig. 2:**
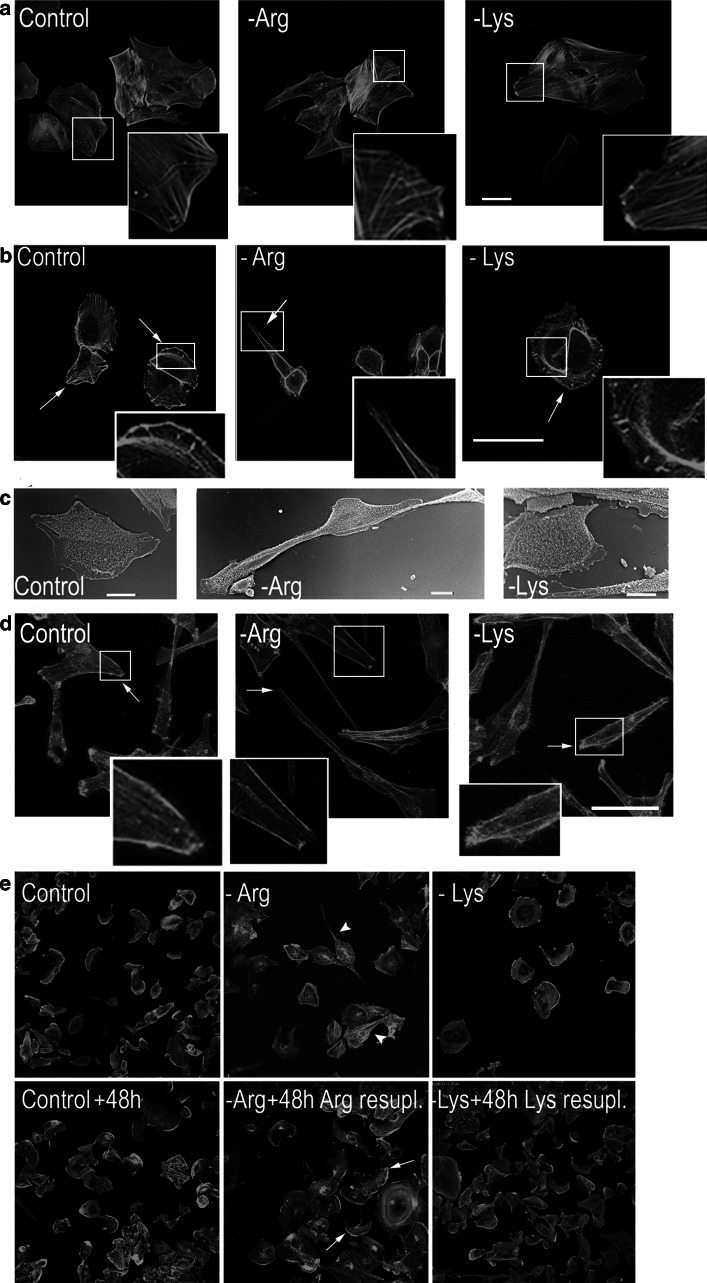
Arginine deprivation affects morphology of glioblastoma but not glia cells. **a**, **b**, **d** Rat glia, U251 and U87 cells stained with Alexa 488-phalloidin, respectively. **c** Micrographs of U251 cells attained with scanning electron microscope. *Insets* in **a**, **b** and **d** ~2–3× magnification of the *marked areas*. *Bars*, in **a**, **b** and **d** 50 μm, and in **c** 10 μm. **e** U251 cells stained with Alexa 488-phalloidin before and after re-supplementation with Arg or Lys up to 0.4 and 0.8 mM concentration, respectively. *Arrows* point to lamellipodia, *arrowheads* point to elongated cells

There were no evident changes in glia cells neither in morphology nor in actin cytoskeleton organization after 48-h arginine or lysine starvation with respect to control cells (Fig. 2a, insets). Of note, though cultured glia cells did not resemble an astrocyte-like morphology they still expressed GFAP (not shown).

However, there was a significant effect of 48-h arginine deprivation on the morphology of the examined glioblastoma cells (Fig. 2b–d), which persisted during 144 h of the treatment (not shown). The majority of arginine-deprived U251 cells became elongated and did not form wide lamellipodium, visible in control and -Lys cells (Fig. 2b, insets). Scanning electron micrographs confirmed prominent changes in morphology and in the leading edge formation in -Arg cells but not in control and -Lys cells (Fig. 2c). Staining for actin filaments revealed less stress fibers and less intensive cortical actin staining in -Arg cells when compared to -Lys and control cells. Similar characteristic changes in microfilament organization were also observed in U87 cells (Fig. 2d, insets).

The observed specific effect of arginine deprivation on cell morphology was reversible since re-supplementation of arginine resulted in fast restoration of U251 cells to the control phenotype (Fig. 2e). The reversion was visible already 3 h after adding arginine (Electronic Supplementary Materials II–IV).

### Arginine deprivation inhibits cell motility

**Fig. 3:**
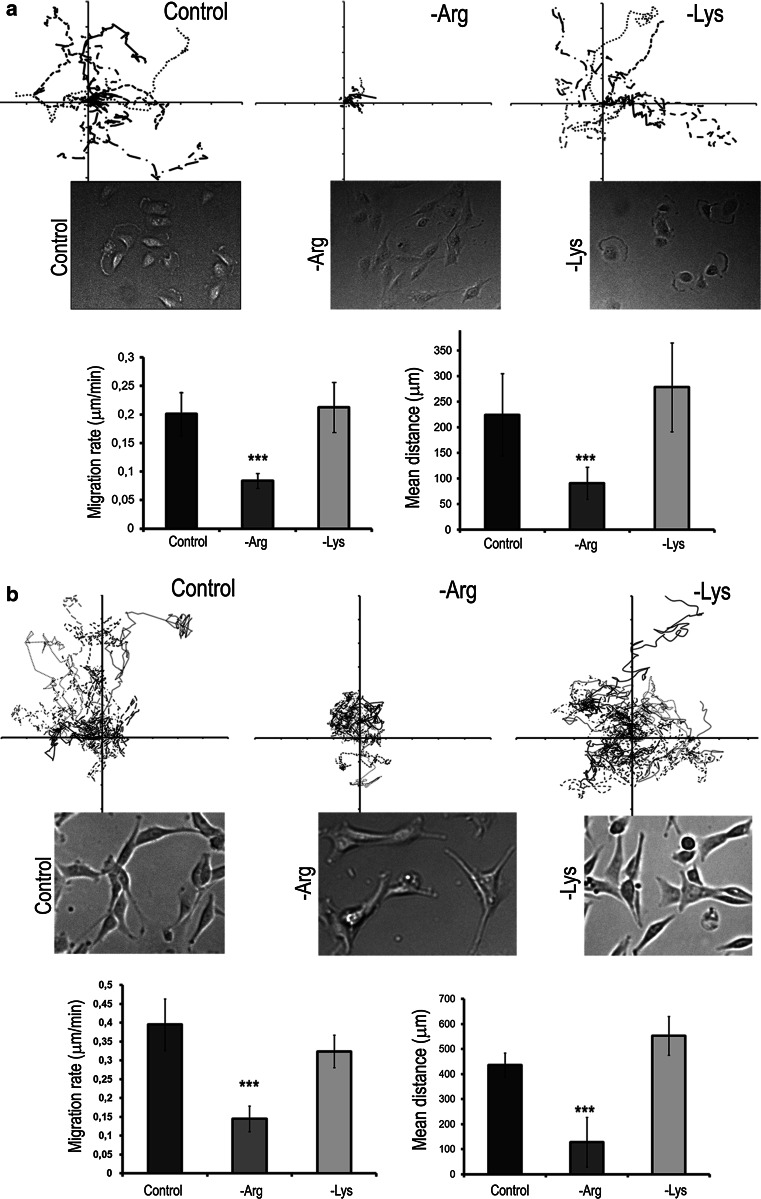
Arginine deprivation impairs cell motility. **a**, **b** Migration tracks of U251 and U87 cells, respectively. *Upper panels* in **a** and **b** tracks of 10 randomly chosen cells; *center panels* images of migrating cells, and *lower panels* values of migration rate and mean distance based on tracks shown in *upper panels*. Values are means ± SD. ***Statistical relevance *p* < 0.001

The changes in the cytoskeleton organization suggest that arginine deprivation could affect glioblastoma cell motility. Therefore, we assessed random cell motility without external chemotactic stimuli using time-lapse microscopy that allowed assessment of migration rate as well as mean distance for individual cells as well as to observe the morphology of motile cells (Fig. 3; Kouvroukoglou et al. 2000). Analysis of 10 randomly chosen cells from each experimental condition revealed that arginine deprivation dramatically decreased the cell speed and mean distance, and concomitantly affected morphology of migrating -Arg cells.

No significant effect of arginine or lysine deprivation on glia cells was observed. Their migration rates in the control, -Arg or -Lys condition were 0.023, 0.020 and 0.021 μm/min, respectively. Also, for 48 h they moved for the distance of 68 μm (control cells), 59 μm (-Arg cells) and 62 μm (-Lys cells).

The inhibitory effect of arginine deprivation on glioblastoma cell motility was confirmed by a wound healing assay (Electronic Supplementary Material V).

### Arginine deprivation impairs cell invasiveness

**Fig. 4:**
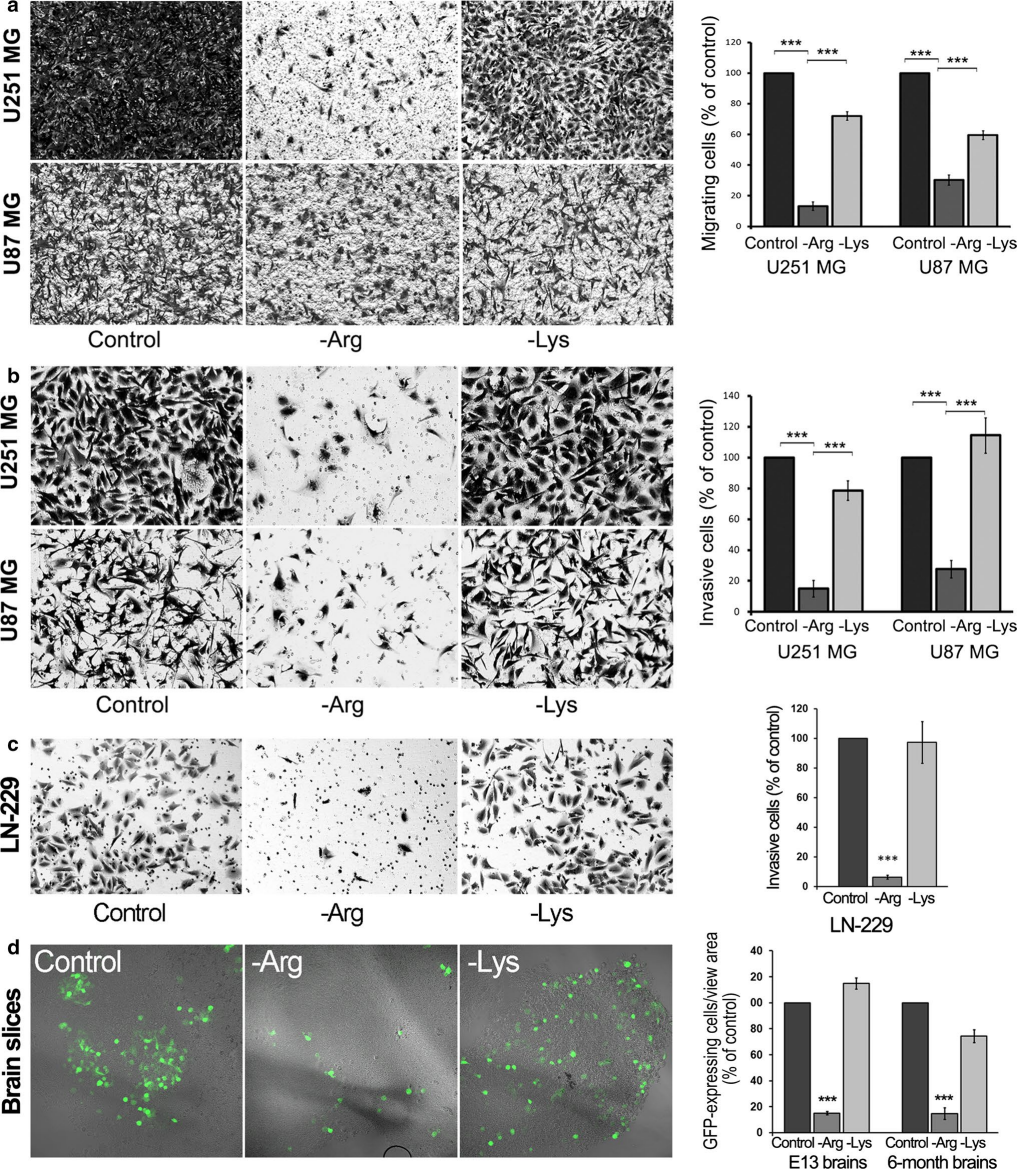
Arginine deprivation impairs cell migration and invasiveness. Transwell filters not covered (**a**), and covered with Matrigel (**b**, **c)** were used for analyses. *Upper* and *lower panels* in **a** and **b** images of U251 and U87 stained cells, respectively, taken on the filter trans side. **c** Images of LN-229 cells, analyzed as in **b**. Analyses were performed for three independent experiments run in duplicates. **d** Images of GFP-expressing U251 cells found within the E13 organotypic brain slice. The* images* represent the confocal 12.3-μm *z*-section of the planar center of brain slices. *Right panel* the quantification of GFP-expressing U251 cells within the confocal center of the slice per view area. The quantitative data in **a**–**d** are presented as % of control. Values are means ± SD. ***Statistical relevance *p* < 0.001

Since arginine deprivation inhibited cell motility, we tested whether arginine deficit could impair cell invasiveness. Experiments were performed using a Transwell filter system in the presence or absence of Matrigel as well as using organotypic brain slices (Fig. 4). As shown in Fig. 4a, -Arg cells passed through the filter less efficiently, as without Matrigel—with respect to control—only ~15 and ~30 % of -Arg U251 and U87 cells, respectively, were found after 6 h on the trans side of the filter. In -Lys conditions ~72 and ~64 % of U87 and U251 cells, respectively, migrated through the filter.

In the presence of Matrigel (Fig. 4b), in -Arg condition only ~15 and ~28 % of U251 and U87 cells, respectively, were found on the filter’s trans side with respect to control conditions. For -Lys conditions these values were ~95 and ~108 % for U251 and U87 cells, respectively.

Similar observation was made for LNB-229 glioblastoma cells that are known to generate invasive tumors (Hlavaty et al. 2011). In -Arg conditions only ~5 % migrated through the Matrigel-coated filter while in -Lys conditions the number was similar to that of control cells (Fig. 4c).

Also, to reveal that arginine deprivation could affect glioblastoma cell invasiveness in vivo we used rat brain organotypic slices of neonatal and adult animals that were uniformly overlayed with GFP-transfected U251 cells, which prior to the experiment were cultured for 24 h in -Arg and -Lys as well as in control media (Fig. 4d). After 24-h co-culture in the examined conditions, the brain slices were washed with PBS and after fixation the amount of fluorescent cells within a slice (i.e. the central planar section) was estimated by means of confocal microscopy. As shown for E13 brain slices (Fig. 4d), -Arg cells did not penetrate the tissue as effectively as the control and -Lys cells. Quantification of images obtained for both E13 and adult brains slices revealed that with respect to the control cells only ~15 % of -Arg cells were found within the slices of both neonatal and mature brains. These numbers were ~115 and ~80 % for -Lys cells.

Thus, the inhibition of glioblastoma cell invasiveness appears to be a specific arginine-related effect.

### Arginine deprivation affects cell adhesion

**Fig. 5:**
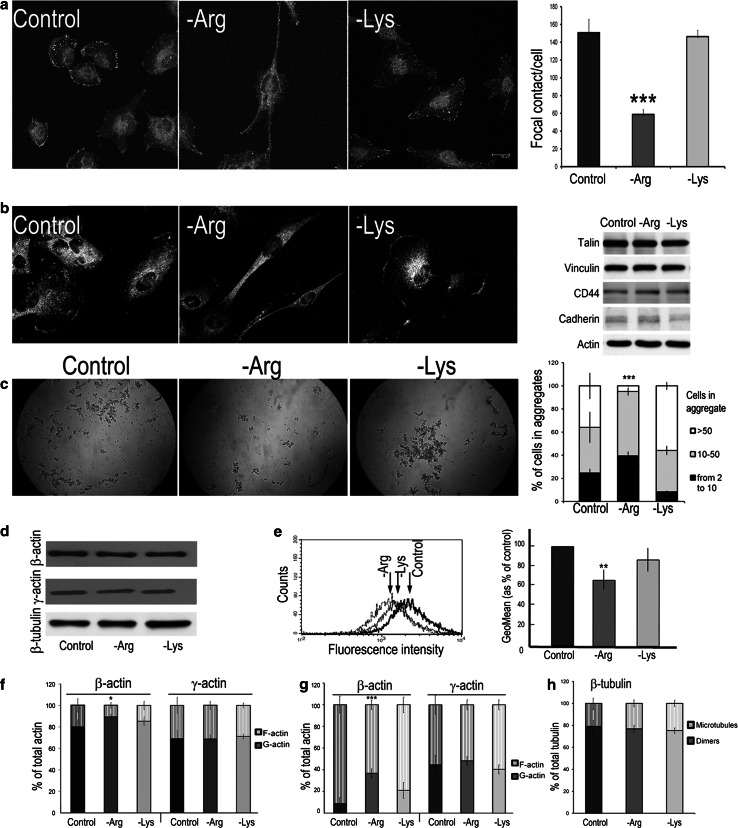
Arginine deprivation affects cell adhesion and β-actin organization. **a**, **b** U251 cells stained with anti-vinculin and anti-talin antibody, respectively. Graph in **a**, quantification of vinculin-containing structures visualized in confocal microscope after 48-h cultivation in the examined conditions; 20 cells were analyzed. *Right panel* in **b** western blot analysis of cell lysates for the presence of talin vinculin, CD44 and E-cadherin in U251 cell after 48-h cultivation in the examined conditions. **c** Assessment of homotypic adhesion by analysis of the formed cell aggregates. **d** Western blots of total cell lysates probed with anti-β- and γ-actin, and anti-β-tubulin antibodies. **e** Flow cytometry analyses of cells stained with Alexa 488-conjugated phalloidin. *Arrows* point to the fluorescence peak of each experimental condition. This is a representative result from four independent experiments. *Right panel* quantitative analysis where 100 % is GeoMean value of control cells. The data in **e** are based on four independent experiments. **f** Quantitative analyses of β- and γ-actin isoforms content in the pellet (F-actin, *upper bar*) and supernatant (G-actin, *lower bar*) fractions. **g** The same as in **f** but in the presence of phalloidin. **h** Quantitative analysis of β-tubulin content performed as in **f**, where lower and upper bars correspond to tubulin dimers and microtubules, respectively. Data in **f**–**h** are from three independent experiments run in duplicates. Data in **a**, **c**, **e** and **f**–**h** are means ± SD. *, **, ***Statistical relevance *p* < 0.05, *p* < 0.01 and *p* < 0.001, respectively

The observed changes in the arginine-deprived glioblastoma cell morphology, migration and invasiveness suggest alterations in cell adhesion. Adhesion was tested as cell attachment to the surface (heterotypic adhesion, Fig. 5a, b) or cell–cell interactions (homotypic adhesion; Fig. 5c).

To assess the adhesive structure formation, U251 cells were immunostained for vinculin and talin, the focal contact markers. Distribution of both proteins in -Arg cells was different from the control and -Lys counterparts as the fluorescence at the cell edges was less intensive (Fig. 5a, b). Quantification of vinculin-stained structures indicates that ~2.5× less adhesion contacts were formed in -Arg cells (starved for 48 h) in comparison with the control and -Lys cells (Fig. 5a, right panel). However, arginine deprivation did not significantly affect the expression of proteins involved in cell adhesion as their levels in -Arg cells did not change with respect to control and -Lys cells as revealed by immunodetection for talin, vinculin, CD44 and E-cadherin (see Fig. 5b, right panel).

For the assessment of clonogenic potential, U251 cells were cultured on soft agar for 24 h, and the amount of aggregates with 2–10, 11–50 and >50 cells was quantified (Fig. 5c). -Arg cells formed significantly fewer aggregates with high cell numbers (~20 % of the >50-cell aggregates relative to control conditions and to 60 % for -Lys cells).

The data indicate that arginine deprivation severely impairs cell adhesion.

### Arginine deprivation affects organization of actin cytoskeleton

The above described arginine deprivation-related effects could be evoked by the observed alterations in actin cytoskeleton organization (see Fig. 2) and/or actin synthesis. We addressed this supposition using U251 cells, which exhibited more pronounced morphological alterations upon arginine deprivation.

Western blotting showed no significant difference in the overall content of β- and γ-actins as well as of β-tubulin in the lysates of -Arg, -Lys and control cells, indicating that arginine deprivation did not affect total actin level (Fig. 5d).

To assess actin filament content, we also employed flow cytometry. Cells were stained with Alexa-conjugated phalloidin and the fluorescence intensity spectra of 10,000 cells were collected. As shown in Fig. 5e, the mean fluorescence intensity (presented as GeoMean) was evidently smaller in -Arg cells than in -Lys and control cells, indicating that -Arg cells contained significantly less actin filaments. As expected, there was practically no difference in the GeoMean values for rat glia cultured in the examined conditions (Electronic Supplementary Material VI).

Since there is no dramatic difference in binding of phalloidin to mammalian actin isoforms (Allen et al. 1996), we performed a sedimentation assay to characterize β- or γ-actins content by Western blotting in the supernatant (containing monomeric G-actin and/or small oligomers) and pellet (containing filamentous F-actin and/or actin bundles). Densitometric analysis of the supernatant and pellet fractions is presented in Fig. 5f. We observed higher content of β-actin in the supernatant fraction of -Arg cells in comparison with -Lys and control cells. Contrary to that, there was no pronounced difference in the γ-actin fraction content. Similar observation was made for lysates, which prior to ultracentrifugation were pre-incubated with phalloidin to prevent possible filament disassembly during sample processing (Fig. 5g).

Analogous analysis of microtubule organization with anti-β-tubulin antibodies revealed no evident difference in the microtubule versus tubulin dimer ratio of samples from -Arg, -Lys and control cells (Fig. 5h).

### Arginine deprivation impairs β-actin arginylation

**Fig. 6:**
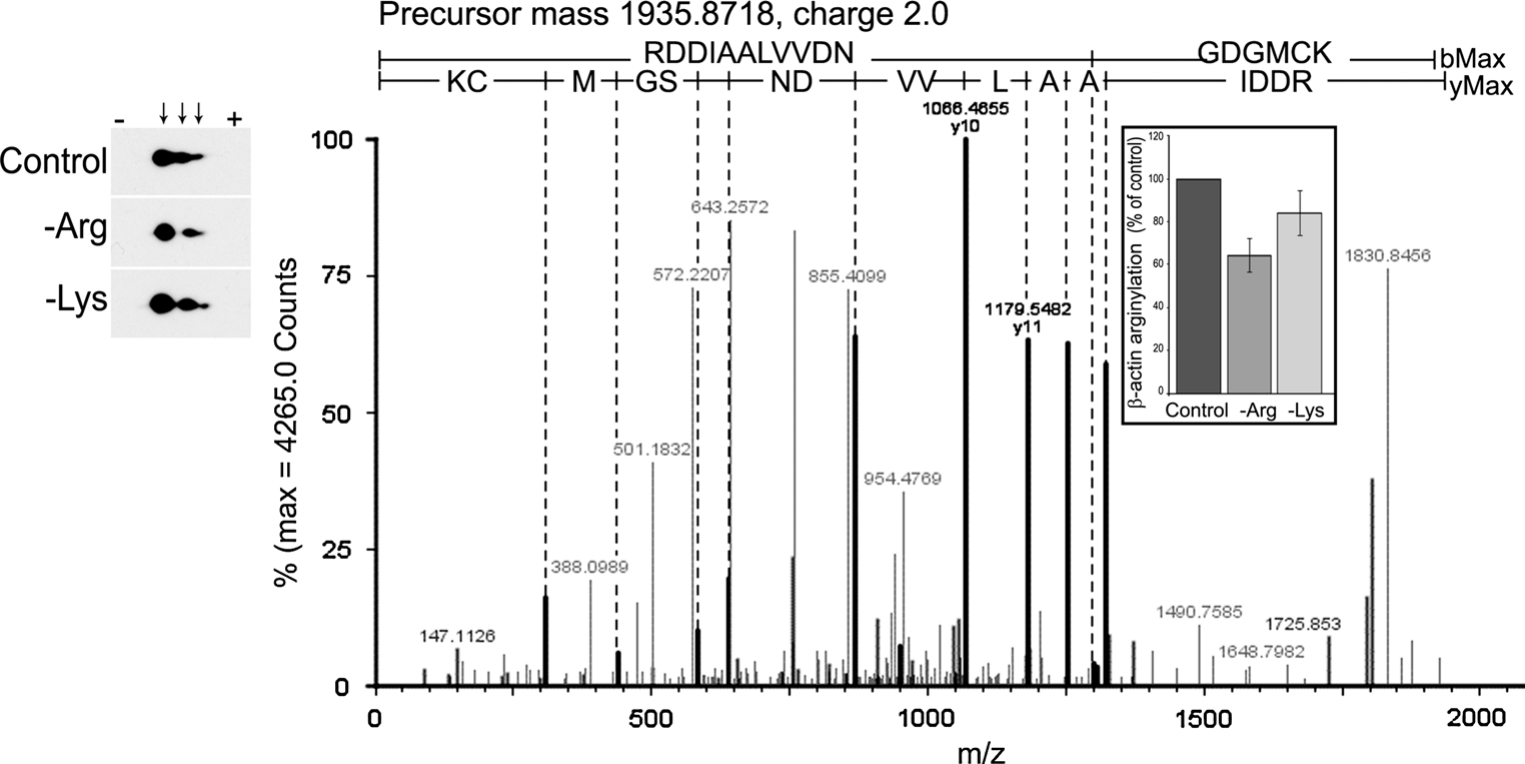
Arginine deprivation impairs β-actin arginylation. **a** 2D-electrophoresis of cell lysates probed with anti-actin antibody. *Arrows* point to major actin isoelectric forms. “−” and “+”, lower and higher pH, respectively. **b** Deconvoluted MS/MS spectrum of the N-terminally arginylated β-actin peptide, with carbamidomethyl modification on cysteine (MH+ 1,935.89 Da, mass error 9.14 ppm). ProteinLynx Global Server software assigned following 15 fragment products of precursor peptide to the observed spectrum (mass error in ppm given in brackets): y2 (−1.4022), y3 (−2.3098), y5 (0.8842), y6 (1.8187), y8 (−0.1555), y10 (0.0295), y11 (−1.0726), y12 (0.4542), y13 (−0.5259), b12 (8.5818), y8-H_2_O (0.1441), y9-H_2_O (−4.6865), y10-H_2_O (24.4993), y7-NH_3_ (5.1754), y13-NH_3_ (11.683). *Inset* in **b**, β-actin arginylation in -Arg and -Lys cells as the % of β-actin arginylation in control cells. Values are means ± SD. ***Statistical relevance *p* < 0.001

The impact of arginine deprivation on cell migration and cytoskeleton organization resembled to some extent the findings in mouse cells with knockdown or treated with inhibitors of arginyl transferase 1 (Ate1), an enzyme responsible for protein arginylation (Saha et al. 2012; Karakozova et al. 2006). This implies that arginine deprivation could affect β-actin arginylation. To verify this hypothesis, we performed 2D electrophoresis and mass spectrometry analysis (Fig. 6).

2D electrophoresis revealed a difference in the actin isoelectric pattern between the analyzed samples (Fig. 6a). Out of three major spots detected by anti-actin antibody in control and -Lys cells, only two with more acidic pI were seen in -Arg cells. This suggests changes in posttranslational modification of actin pattern, possibly due to impairment in β-actin arginylation.

MS^E data analysis with ProteinLynx Global Server 2.4 software allowed identification of arginylated N-terminal peptide. With this approach, we found Arg residue on the N-terminus of Asp2 and not on Asp3 of the β-actin sequence (RDDIAALVVDNGSGMCK), and its assignment to a mass peak on chromatogram based on the measured parent peptide mass and its corresponding fragment mass spectrum (Fig. 6b). The peptide coverage for β-actin molecule was between 78 and 88 % depending on the run.

To compare the extent of N-terminal protein arginylation between the control and -Arg or -Lys samples, the chromatograms for masses (chosen ion intensities as measured by the instrument, versus scan numbers) of non-fragmented arginylated N-terminal peptide and for five randomly selected peptides from the digest were integrated and the arginylation extent was established as described in Methods section. The extent of actin arginylation for control samples (*n* = 4) was set as 100 %, and for -Arg samples was calculated as 64 ± 8 % (*n* = 4) and for -Lys samples as 84 ± 11 % (*n* = 3) (Fig. 6b, inset). Therefore these data confirm a more pronounced relative decrease in arginylated β-actin in the arginine-deprived cells.

## Discussion

A number of studies evaluated arginine deprivation-based strategy as potential anti-cancer therapy, which allows to control growth of tumors deficient in arginine biosynthesis (reviewed in Delage et al. 2010; Feun et al. 2008; Wheatley 2004; Yoon et al. 2013). Many cultured tumor cells are highly sensitive to arginine deprivation, an essential amino acid for malignant cells in vitro (Bobak et al. 2010), and often exhibit progressive decrease in viability and signs of apoptosis (Delage et al. 2010). Such response, however, does not necessarily translates to 3D cultures of tumor cells (Vynnytska-Myronovska et al. 2012), or to in vivo, as revealed by clinical trials with recombinant arginine-degrading enzymes (Glazer et al. 2010; Yang et al. 2010; Yau et al. 2013).

Surprisingly up to now there were no reports on the mechanisms of arginine deprivation on malignant cells metastatic potential. Also, effect of arginine deprivation on brain physiology was not studied, though its prominent role in brain development and degeneration was shown (Yi et al. 2009). However, if the problem of in vivo arginine conversion from its precursors, primarily citrulline, is resolved (Syed et al. 2013), brain cancers can be potential objects of arginine deprivation-based therapy because such a metabolic approach is not dependent on permeability of the brain-blood barrier, which is often a concern for brain tumors chemotherapy.

We report for the first time that arginine deprivation strongly impairs motility, migration and invasiveness as well as hetero- and homotypic adhesion of human glioblastoma cell lines. We showed that these effects were associated with changes in the actin cytoskeleton organization that could result from depletion of β-actin arginylation. The observed phenomena were arginine-specific as deprivation for lysine, also a positively charged essential amino acid, caused significantly milder effects. Lysine deprivation was chosen as an experimental control to discern between the general effects of a single essential amino acid starvation and those specific only for arginine deprivation.

Although starvation for arginine abrogated cell growth, decreased in a time-dependent manner cell viability and altered cytoskeletal organization; these effects were reversed upon restoring optimal growth conditions by arginine re-supplementation. Therefore, being relatively resistant to arginine deprivation, three glioblastoma cell lines served in our hands as useful and informative models for elucidating and dissecting possible specific effects of arginine withdrawal relevant to metastatic potential of malignant cells. Importantly, we did not observe any evident effect of arginine or lysine starvation on primary rat glia cell morphology, migration and microfilament organization.

Impairment of migration and adhesion is predominantly due to aberrations in actin cytoskeleton organization (Pollard and Cooper 2009). Using several independent techniques, we demonstrated that changes observed in glioblastoma cell morphology and behavior under arginine deprivation were associated with actin cytoskeleton remodeling but not with overall actin level. Also, we showed that arginine deprivation-evoked decrease in filament content concerned β-actin but not γ-actin isoform, and demonstrated that arginine deprivation diminished the extent of β-actin arginylation, a posttranslational modification mediated by arginyltransferase (Ate1). Arginylation of this actin isoform was already shown to be crucial for cell migration and cardiomyocyte contractility (Terman and Kashina 2013; Saha et al. 2012; Kurosaka et al. 2012). It is plausible that arginylation of other cytoskeletal proteins could be also affected in -Arg cells since as it was previously reported numerous actin-binding proteins, including talin had been found to be arginylated (Saha et al. 2010; Wong et al. 2007; Zhang et al. 2012). Thus it is possible that the observed defects in adhesive structure formation could be also related to arginylation of talin and/or other protein(s) involved in adhesion complex formation and cell–cell interactions (Wong et al. 2007; Zhang et al. 2012).

Summarizing, we showed for the first time that arginine deprivation-dependent effects on glioblastoma cell morphology, adhesion, migration and invasiveness were associated with specific changes in actin cytoskeleton organization caused by decrease in β-actin arginylation. Since these effects concerned malignant but not untransformed glia cells, one may consider arginine deprivation-based strategy, possibly rationally designed combinational regimen for the control and treatment of brain tumors. Further studies are certainly needed to fully understand molecular mechanisms underlying these effects, and whether they also take place in other tumors. Recent findings that intrinsic amino acid catabolism is involved in determining glioma aggressiveness in vivo (Tönjes et al. 2013) and that some brain tumors are deficient in arginine biosynthesis (Syed et al. 2013) draw further interest to the development of metabolic therapies against these highly malignant tumors. However, it has also to be experimentally addressed whether and how prolonged arginine deprivation affects functioning of immune system and brain physiology, in particular. This is an important aspect especially in the light of the data indicating that depletion of arginine has a differential impact on the activation and functions of T cells and macrophages (Choi et al. 2009).

## Electronic supplementary material

Below is the link to the electronic supplementary material.

Supplementary material 1 (DOC 156 kb)

Supplementary material 2 (DOC 21 kb)

Supplementary material 3 (AVI 4026 kb)

Supplementary material 4 (AVI 3082 kb)

Supplementary material 5 (DOC 651 kb)

Supplementary material 6 (TIFF 1451 kb)

Supplementary material 7 (AVI 4130 kb)

## References

[CR1] Agrawal V, Alpini SE, Stone EM, Frenkel EP, Frankel AE (2012). Targeting methionine auxotrophy in cancer: discovery and exploration. Expert Opin Biol Ther.

[CR2] Albini A, Iwamoto Y, Kleinman HK, Martin GR, Aaronson SA, Kozlowski JM, McEwan RN (1987). A rapid in vitro assay for quantitating the invasive potential of tumor cells. Cancer Res.

[CR3] Allen PG, Shuster CB, Käs J, Chaponnier C, Janmey PA, Herman IM (1996). Phalloidin binding and rheological differences among actin isoforms. Biochemistry.

[CR4] Allen MD, Luong P, Hudson C (2014). Prognostic and therapeutic impact of argininosuccinate synthetase 1 control in bladder cancer as monitored longitudinally by PET imaging. Cancer Res.

[CR5] Bobak YP, Vynnytska BO, Kurlishchuk YV, Sibirny AA, Stasyk OV (2010). Cancer cell sensitivity to arginine deprivation in vitro is not determined by endogenous levels of arginine metabolic enzymes. Cell Biol Int.

[CR6] Bowles TL, Kim R, Galante J, Parsons CM, Virudachalam S, Kung HJ, Bold RJ (2008). Pancreatic cancer cell lines deficient in argininosuccinate synthetase are sensitive to arginine deprivation by arginine deiminase. Int J Cancer.

[CR7] Brandes AA (2007). State-of-the-art treatment of high-grade brain tumors. Semin Oncol.

[CR8] Bregier C, Krzemień-Ojak L, Włoga D (2013). PHLP2 is essential and plays a role in ciliogenesis and microtubule assembly in *Tetrahymena thermophila*. J Cell Physiol.

[CR9] Choi BS, Martinez-Falero IC, Corset C, Munder M, Modolell M, Müller I, Kropf P (2009). Differential impact of l-arginine deprivation on the activation and effector functions of T cells and macrophages. J Leukoc Biol.

[CR10] Covini D, Tardito S, Bussolati O, Chiarelli LR, Pasquetto MV, Digilio R, Valentini G, Scotti C (2012). Expanding targets for a metabolic therapy of cancer: l-asparaginase. Recent Pat Anticancer Drug Discov.

[CR11] Delage B, Fennell DA, Nicholson L, McNeish I, Lemoine NR, Crook T, Szlosarek PW (2010). Arginine deprivation and argininosuccinate synthetase expression in the treatment of cancer. Int J Cancer.

[CR12] Dillon BJ, Prieto VG, Curley SA, Ensor CM, Holtsberg FW, Bomalaski JS, Clark MA (2004). Incidence and distribution of argininosuccinate synthetase deficiency in human cancers: a method for identifying cancers sensitive to arginine deprivation. Cancer.

[CR13] Drozak J, Chrobok L, Poleszak O, Jagielski AK, Derlacz R (2013). Molecular identification of carnosine N-methyltransferase as chicken histamine N-methyltransferase-like protein (hnmt-like). PLoS ONE.

[CR14] Feun L, You M, Wu CJ, Kuo MT, Wangpaichitr M, Spector S, Savaraj N (2008). Arginine deprivation as a targeted therapy for cancer. Curr Pharm Des.

[CR15] Glazer ES, Piccirillo M, Albino V (2010). Phase II study of pegylated arginine deiminase for nonresectable and metastatic hepatocellular carcinoma. J Clin Oncol.

[CR16] Hlavaty J, Jandl G, Liszt M, Petznek H, König-Schuster M, Sedlak J, Egerbacher M, Weissenberger J, Salmons B, Günzburg WH, Renner M (2011). Comparative evaluation of preclinical in vivo models for the assessment of replicating retroviral vectors for the treatment of glioblastoma. J Neurooncol.

[CR17] Karakozova M, Kozak M, Wong CC (2006). Arginylation of β-actin regulates actin cytoskeleton and cell motility. Science.

[CR18] Kim RH, Coates JM, Bowles TL (2009). Arginine deiminase as a novel therapy for prostate cancer induces autophagy and caspase-independent apoptosis. Cancer Res.

[CR19] Kouvroukoglou S, Dee KC, Bizios R, McIntire LV, Zygourakis K (2000). Endothelial cell migration on surfaces modified with immobilized adhesive peptides. Biomaterials.

[CR20] Kurosaka S, Leu NA, Pavlov I (2012). Arginylation regulates myofibrils to maintain heart function and prevent dilated cardiomyopathy. J Mol Cell Cardiol.

[CR21] Majewski L, Sobczak M, Wasik A, Skowronek K, Rędowicz MJ (2011). Myosin VI in PC12 cells play important roles in cell migration and proliferation but not in catecholamine secretion. J Muscle Res Cell Motil.

[CR22] Morris SM (2007). Arginine metabolism: boundaries of our knowledge. J Nutr.

[CR23] Morrow K, Hernandez CP, Raber P (2013). Anti-leukemic mechanisms of pegylated arginase I in acute lymphoblastic T-cell leukemia. Leukemia.

[CR24] Peickert S, Waurig J, Dittfeld C (2012). Rapid re-expression of CD133 protein in colorectal cancer cell lines in vitro and in vivo. Lab Invest.

[CR25] Pollard TD, Cooper JA (2009). Actin, a central player in cell shape and movement. Science.

[CR26] Saha S, Mundia MM, Zhang F (2010). Arginylation regulates intracellular actin polymer level by modulating actin properties and binding of capping and severing proteins. Mol Biol Cell.

[CR27] Saha S, Wang J, Buckley B, Wang Q, Lilly B, Chernov M, Kashina A (2012). Small molecule inhibitors of arginyltransferase regulate arginylation-dependent protein degradation, cell motility, and angiogenesis. Biochem Pharmacol.

[CR28] Scott L, Lamb J, Smith S, Wheatley DN (2000). Single amino acid (arginine) deprivation: rapid and selective death of cultured transformed and malignant cells. Br J Cancer.

[CR29] Shevchenko A, Wilm M, Vorm O, Mann M (1996). Mass spectrometric sequencing of proteins silver-stained polyacrylamide gels. Anal Chem.

[CR30] Shevchenko A, Tomas H, Havlis J, Olsen JV, Mann M (2006). In-gel digestion for mass spectrometric characterization of proteins and proteomes. Nat Protocol.

[CR31] Stoppini L, Buchs PA, Muller D (1991). A simple method for organotypic cultures of nervous tissue. J Neurosci Methods.

[CR32] Syed N, Langer J, Janczar K (2013). Epigenetic status of argininosuccinate synthetase and argininosuccinate lyase modulates autophagy and cell death in glioblastoma. Cell Death Dis.

[CR33] Terman JR, Kashina A (2013). Post-translational modification and regulation of actin. Curr Opin Cell Biol.

[CR34] Tönjes M, Barbus S, Park YJ (2013). BCAT1 promotes cell proliferation through amino acid catabolism in gliomas carrying wild-type IDH1. Nat Med.

[CR35] Vynnytska-Myronovska B, Bobak Y, Garbe Y, Dittfeld C, Stasyk O, Kunz-Schughart LA (2012). Single amino acid arginine starvation efficiently sensitizes cancer cells to canavanine treatment and irradiation. Int J Cancer.

[CR36] Wheatley DN (2004). Controlling cancer by restricting arginine availability—arginine-catabolizing enzymes as anticancer agents. Anticancer Drugs.

[CR37] Wheatley DN, Kilfeather R, Stitt A, Campbell E (2005). Integrity and stability of the citrulline-arginine pathway in normal and tumour cell lines. Cancer Lett.

[CR38] Wong CC, Xu T, Rai R, Bailey AO, Yates JR, Wolf YI, Zebroski H, Kashina A (2007). Global analysis of posttranslational protein arginylation. PLoS Biol.

[CR39] Yang TS, Lu SN, Chao Y (2010). A randomised phase II study of pegylated arginine deiminase (ADI-PEG 20) in Asian advanced hepatocellular carcinoma patients. Br J Cancer.

[CR40] Yau T, Cheng PN, Chan P, Chan W, Chen L, Yuen J, Pang R, Fan ST, Poon RT (2013). Aphase 1 dose-escalating study of pegylated recombinant human arginase 1 (Peg-rhArg1) in patients with advanced hepatocellular carcinoma. Invest NewDrugs.

[CR41] Yi J, Horky LL, Friedlich AL, Shi Y, Rogers JT, Huang X (2009). l-arginine and Alzheimer’s disease. Int J Clin Exp Pathol.

[CR42] Yoon CY, Shim YJ, Kim EH, Lee JH, Won NH, Kim JH, Park IS, Yoon DK, Min BH (2007). Renal cell carcinoma does not express argininosuccinate synthetase and is highly sensitive to arginine deprivation via arginine deiminase. Int J Cancer.

[CR43] Yoon JK, Frankel AE, Feun LG, Ekmekcioglu S, Kim KB (2013). Arginine deprivation therapy for malignant melanoma. Clin Pharmacol.

[CR44] Zawadzka M, Kaminska B (2003). Immunosuppressant FK506 affects multiple signaling pathways and modulates gene expression in astrocytes. Mol Cell Neurosci.

[CR45] Zhang F, Saha S, Kashina A (2012). Arginylation-dependent regulation of a proteolytic product of talin is essential for cell-cell adhesion. J Cell Biol.

