# Supplementary material for: Arginine deprivation affects glioblastoma cell adhesion, invasiveness and actin cytoskeleton organization by impairment of β-actin arginylation
Source: Amino Acids. 2014 Nov 2;47(1):199–212. doi: 10.1007/s00726-014-1857-1 (PMC4282698; doi:10.1007/s00726-014-1857-1)
Supplement: Supplementary file 1 — Supplementary material 1 (DOC 156 kb) [file 726_2014_1857_MOESM1_ESM.doc]

**Pavlyk et al. Supplementary material I**

***Methods:***

***Immunoblotting.*** Protein extracts (~20 μg) of cells grown in -Arg, -Lys and control conditions were separated using 12% SDS-PAGE, and then transferred to a nitrocellulose membrane (Bio-Rad, USA). Polyclonal antibody against PARP (Cell Signaling, USA) and monoclonal antibody against β-actin (Sigma-Aldrich, USA) were used at 1:1000 and 1:5000 dilutions, respectively, and then detected with anti-rabbit and anti-mouse antibodies (diluted 1:10,000) conjugated with horse radish peroxidase. The reaction was developed using ECL according to the manufacturer’s instructions (Pierce, USA). Protein concentration was determined by Bio-Rad protein assay reagent (Bio-Rad, USA).

***Apoptosis assay.*** The quantification of apoptosis was determined by flow cytometry using the Annexin V- Apoptosis Detection Kit according to the manufacturer's instructions (BD Pharmingen, BD Bioscience, USA). Briefly, U251 cells after growth for 48 h in control, -Arg and -Lys media were washed with PBS, suspended in 1x binding buffer and then added to PE Annexin V and 7-AAD. The samples were then analyzed using FACScalibur (BD Bioscience, USA) using CellQuest Pro analysis software (Becton Dickinson, USA). Control cells were treated with doxorubicin at concentration was 10 μmol/L

***
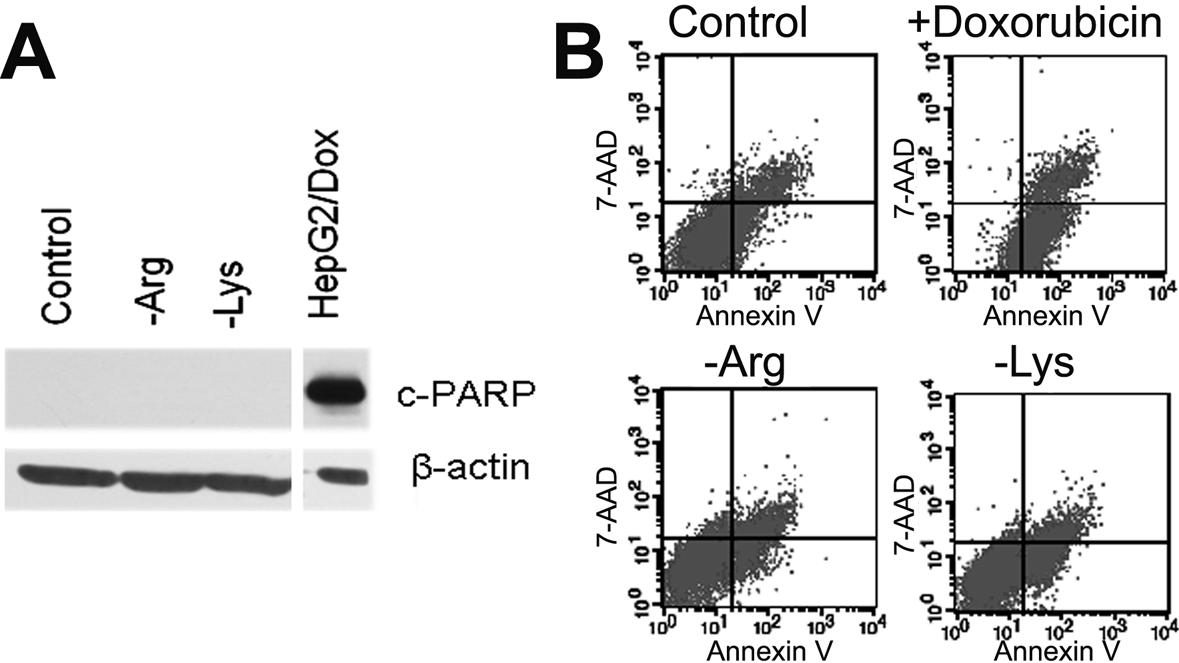
***

Supplementary Figure I. Arginine deprivation does not induce apoptosis. A. There was no detection of the degradation product of PARP (c-PARP) in the lysates of U251 MG cultivated in control, -Arg and -Lys conditions. Lysates of HepG2 cells cultivated in the presence of xxx mg/ml of doxorubicin served as the positive control. B. Annexin-V apoptosis test was performed for U251 MG cells cultivated in the control, -Arg and -Lys as well as in the presence of doxorubicin according to the manufacturer's instructions.

The data provided by means of western blotting and flow cytometry (Supplementary Figure I) indicate that neither arginine nor lysine deprivation induce U251 MG cell apoptosis.
