# Supplementary material for: Arginine deprivation affects glioblastoma cell adhesion, invasiveness and actin cytoskeleton organization by impairment of β-actin arginylation
Source: Amino Acids. 2014 Nov 2;47(1):199–212. doi: 10.1007/s00726-014-1857-1 (PMC4282698; doi:10.1007/s00726-014-1857-1)
Supplement: Supplementary file 2 — Supplementary material 2 (DOC 21 kb) [file 726_2014_1857_MOESM2_ESM.doc]

**Pavlyk et al. Supplementary material II-IV: description of video materials**

*Video 1* (Control cells)

Time lapse images of control U251 MG glioblastoma cells.

Images represent motile control cells producing ruffles and wide lamellae.

The recording was performed for 17 h.

*Video 2* (-Arg +48h Arg resuppl.)

Time lapse images of motile U251 MG glioblastoma cells cultivated for 48 h in -Arg conditions and then re-supplemented with arginine.

The recording was performed for 17 h. The cells start to form wide lamellae already after ~3 h upon re-supplementation.

*Video 3* (-Lys +48h Lys resuppl.)

Time lapse images of motile U251 MG glioblastoma cells cultivated for 48 h in -Lys conditions and then re-supplemented with lysine.

Lys-deprived cells resemble the control cells regardless the presence or absence of lysine.

The recording was performed for 17 h.
