# Supplementary material for: Arginine deprivation affects glioblastoma cell adhesion, invasiveness and actin cytoskeleton organization by impairment of β-actin arginylation
Source: Amino Acids. 2014 Nov 2;47(1):199–212. doi: 10.1007/s00726-014-1857-1 (PMC4282698; doi:10.1007/s00726-014-1857-1)
Supplement: Supplementary file 5 — Supplementary material 5 (DOC 651 kb) [file 726_2014_1857_MOESM5_ESM.doc]

**Pavlyk et al. Supplementary material V**

***Method:***

***Wound Healing Assay.*** Cells (3×10^4^ cells/well) were allowed to form a confluent monolayer in a 6-well-plate, and then the layer was scratched using 200-μL tip and photographed with 10 x objective of the inverted Zeiss LSM780 microscope equipped with environmental chamber. The wound area was measured using ImageJ software, and the rate of cell migration was calculated as the percentage of the remaining cell free area compared with the initial wound area.

**
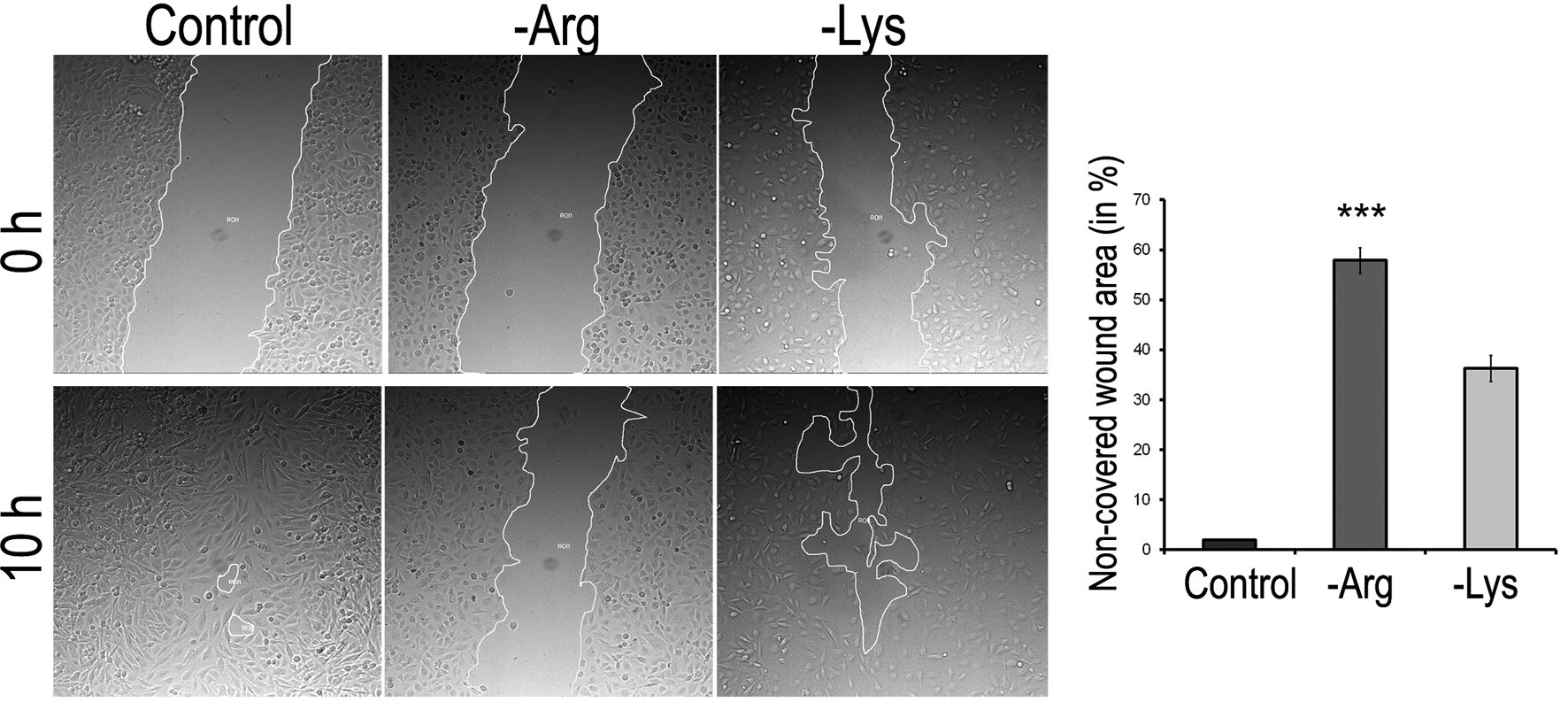
**

Suppl. Figure V. Arginine deprivation impairs cell motility examined by the wound healing assay. Images of the same scratch areas (marked by white solid line) were taken at 0 and 10 h using optical microscope, where 0 is the time of recording initiation. Migration presented as % of the scratch area non-covered by the cells after 10 h in respect to 0 h. These are representative images and the data from three independent experiments.

Migration of U251 MG cells was assessed by the wound healing assay (Suppl. Figure V). Cells cultivated for 48 h up to confluence were photographed after additional 10 h (Fig. 4A) and the quantitative analysis of the wound area (not covered by the cells) revealed that it was substantially bigger in -Arg cells (~55%) than in -Lys (~40%) in also non-proliferative cells. The data indicate that arginine deprivation impairs glioblastoma cell migration.
