# Supplementary figures and images for: Arginine deprivation affects glioblastoma cell adhesion, invasiveness and actin cytoskeleton organization by impairment of β-actin arginylation
Source: Amino Acids. 2014 Nov 2;47(1):199–212. doi: 10.1007/s00726-014-1857-1 (PMC4282698; doi:10.1007/s00726-014-1857-1)

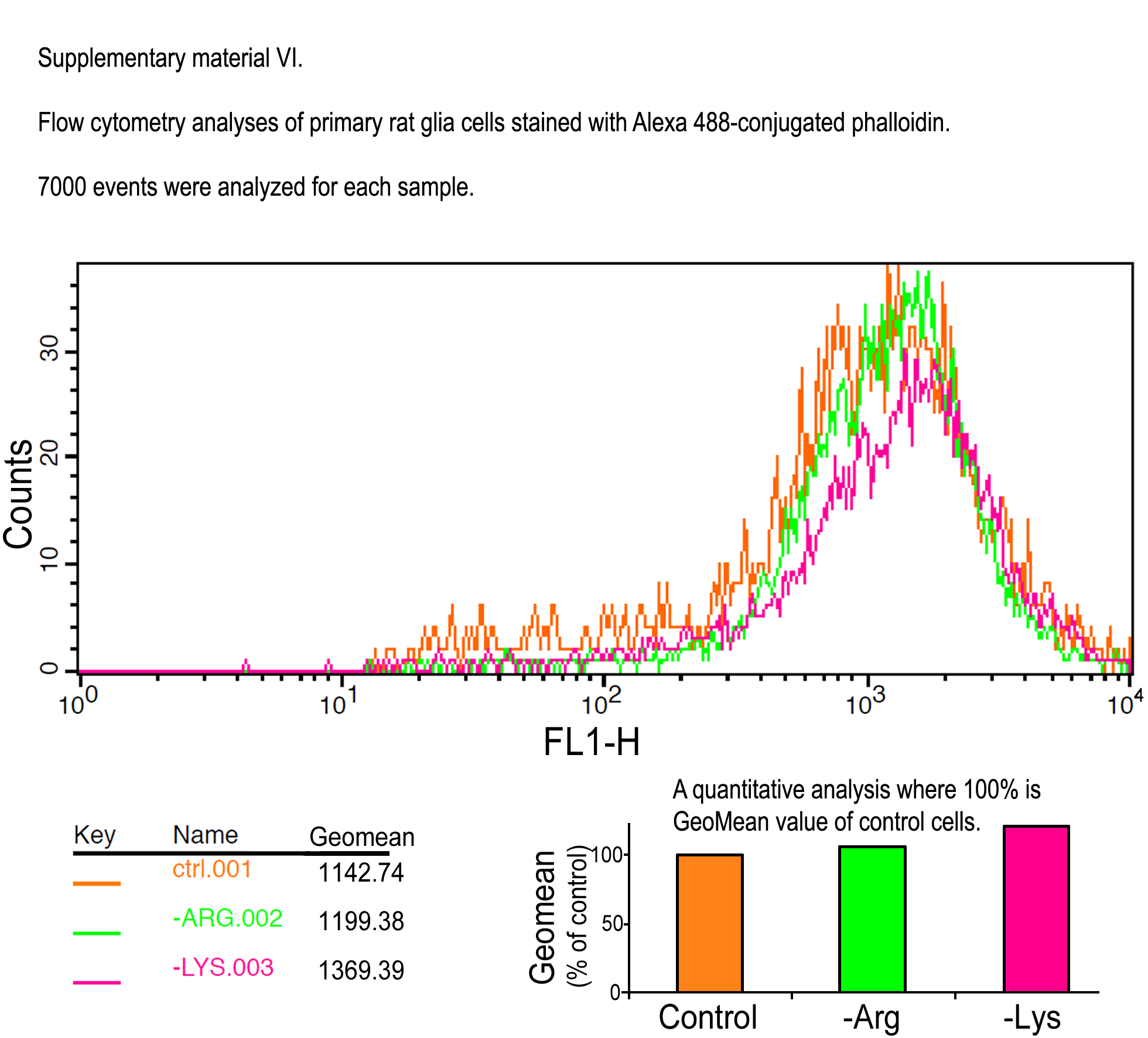

Supplement: Supplementary file 6 — Supplementary material 6 (TIFF 1451 kb) [file 726_2014_1857_MOESM6_ESM.tif]
